# Stimulation of antiviral cellular immune responses by therapeutic vaccination of HIV-1-infected patients with dendritic cells transfected with *gag*, *tat*, *rev* and *nef* mRNA

**DOI:** 10.1186/1742-4690-8-S2-P76

**Published:** 2011-10-03

**Authors:** Ellen Van Gulck, Viggo F Van Tendeloo, Erika Vlieghe, Marc Vekemans, Ann Van de Velde, Evelien Smits, Sébastien Anguilie, Nathalie Cools, Barbara Stein, Griet Nijs, Herman Goossens, Liesbet Mertens, Winni De Haes, Céline Merlin, Derek Atkinson, Johnsson Wong, Eric Florence, Guido Vanham, Zwi N Berneman

**Affiliations:** 1 Department of Biomedical Sciences, Division of Microbiology, Virology Unit, Institute of Tropical Medicine, Antwerp, Belgium; 2 Vaccine and Infectious Disease Institute, Faculty of Medicine, University of Antwerp, Antwerp, Belgium; 3 Center for Cell Therapy and Regenerative Medicine (CCRG) and Division of Hematology, Antwerp University Hospital (UZA), Edegem, Belgium; 4 Department of Clinical Sciences, Medical Service, HIV and STD Unit, Institute of Tropical Medicine, Antwerp, Belgium; 5 Department of Medicine, Massachusetts General Hospital, Harvard Medical School, Boston, MA, USA; 6 Faculty of Pharmaceutical, Biomedical Sciences and Veterinary Sciences, University of Antwerp, Antwerp and Faculty of Medicine and Pharmacy, Free University of Brussels (VUB), Brussels, Belgium

## Background

In an attempt to raise protective antiviral immunity, dendritic cell (DC) immunotherapy was evaluated in 6 adults infected with human immunodeficiency virus (HIV)-1 and stable under antiretroviral therapy (HAART).

## Methods

Autologous monocyte-derived DC electroporated with mRNA encoding Gag and TatRevNef fusion protein were injected 4 times at 4 weeks interval, while patients remained on HAART. Feasibility, safety and immunogenicity were investigated.

## Results

DC vaccine preparation and administration was successful in all patients and only mild adverse events such as skin reactions were seen. DC vaccination induced immune responses that have been reported to be related to control of HIV-1 replication. There was a significant increase post-as compared to pre-DC vaccination, in magnitude - in particular to Gag – and breadth of HIV-1-specific interferon (IFN)-γ response and T-cell proliferation. Breadth of IFN-γ response and T-cell proliferation were correlated with both CD4+ and CD8+ polyfunctional T-cell responses. Importantly, DC vaccination induced or increased the capacity of autologous CD8+ T-cells to suppress superinfection of CD4+ T-cells with the vaccine-related IIIB virus and to a lesser extent with other HIV-1 strains. This CD8+ T-cell-mediated HIV-1-inhibitory activity was correlated with increased breadth of Gag-specific IFN-γ response, indicative of improved control of HIV replication. These features are indicative of improved virus control.

## Conclusion

Therapeutic immunization of patients stable under HAART with DC electroporated with mRNA encoding HIV-1 antigens is safe and was successful in raising antiviral cellular immune responses, including effector CD8+T-cells with inhibitory activity towards infection of CD4+T-cells with a vaccine-homologous HIV strain.

